# vMMN for schematic faces: automatic detection of change in emotional expression

**DOI:** 10.3389/fnhum.2013.00714

**Published:** 2013-10-28

**Authors:** Kairi Kreegipuu, Nele Kuldkepp, Oliver Sibolt, Mai Toom, Jüri Allik, Risto Näätänen

**Affiliations:** ^1^Department of Experimental Psychology, Institute of Psychology, University of TartuTartu, Estonia; ^2^Doctoral School of Behavioural, Social and Health SciencesTartu, Estonia; ^3^Estonian Academy of SciencesTallinn, Estonia; ^4^Center of Integrative Neuroscience, University of AarhusAarhus, Denmark; ^5^Cognitive Brain Research Unit, Institute of Behavioural Sciences, University of HelsinkiHelsinki, Finland

**Keywords:** visual mismatch negativity, optimal design, oddball design, angry schematic face, happy schematic face

## Abstract

Our brain is able to automatically detect changes in sensory stimulation, including in vision. A large variety of changes of features in stimulation elicit a deviance-reflecting event-related potential (ERP) component known as the mismatch negativity (MMN). The present study has three main goals: (1) to register vMMN using a rapidly presented stream of schematic faces (neutral, happy, and angry; adapted from Öhman etal., 2001); (2) to compare elicited vMMNs to angry and happy schematic faces in two different paradigms, in a traditional oddball design with frequent standard and rare target and deviant stimuli (12.5% each) and in an version of an optimal multi-feature paradigm with several deviant stimuli (altogether 37.5%) in the stimulus block; (3) to compare vMMNs to subjective ratings of valence, arousal and attention capture for happy and angry schematic faces, i.e., to estimate the effect of affective value of stimuli on their automatic detection. Eleven observers (19–32 years, six women) took part in both experiments, an oddball and optimum paradigm. Stimuli were rapidly presented schematic faces and an object with face-features that served as the target stimulus to be detected by a button-press. Results show that a vMMN-type response at posterior sites was equally elicited in both experiments. Post-experimental reports confirmed that the angry face attracted more automatic attention than the happy face but the difference did not emerge directly at the ERP level. Thus, when interested in studying change detection in facial expressions we encourage the use of the optimum (multi-feature) design in order to save time and other experimental resources.

## INTRODUCTION

We are built to perform sparingly. For example, we do not expend perceptual resources at a stable (i.e., highly predictable) level of stimulation. The situation is different with changes in stimulation. The change could be a possible signal of an error, challenge, danger or just a need to react, which triggers a specific neuronal response in the brain, a mismatch negativity (MMN; Näätänen et al., 1978; Näätänen and Michie, 1979). The MMN is a change detection component of the event-related potentials (ERPs) curve that is obtained when the averaged ERP for the frequent standard stimulus is subtracted from that for the rare deviant stimulus. Since its discovery in an auditory modality in 1978, the MMN has been reported to reflect any discriminable changes (see Näätänen et al., 2007 for a recent review). Further support for the view of the MMN as a general reflection of deviance detection in the brain comes from studies of different modalities – vision (e.g., Czigler et al., 2002; Berti and Schröger, 2004; Lorenzo-López et al., 2004; Kremláček, 2006; Astikainen and Hietanen, 2009; Stefanics et al., 2012, etc., see a review in Czigler, 2007), touch (e.g., Kekoni et al., 1997; Shinozaki et al., 1998; Astikainen et al., 2001) and olfaction (e.g., Krauel et al., 1999). Thus, the MMN can be viewed as the most general cortical indicator of an unfulfilled prediction.

Establishing the MMN in vision (i.e., vMMN) still took some time and effort and the main reason is obviously the different relation between vision and attention. One of the necessary properties of the MMN is its independence of attention (Näätänen, 1992; Maekawa et al., 2005) – it is even better observed in a passive (ignore) condition than in an attended condition. It is, of course, much harder to achieve an attention-free testing situation in vision than in hearing due to eyeblinks and directedness of the sight. Maekawa et al. (2005) stated that for vision, it is only possible to have participants engaged with a task when one keeps the stimuli under investigation absolutely irrelevant. No more rigorous guidelines have been given. Even perfect performance in the auditory overt task does not guarantee that there are not enough attention resources for visual stimuli. This doubt has also been recently expressed by Stefanics et al. (2012). Researchers have solved the problem of attention control by using different practices. Some have used a story listening with a later check about its contents (e.g., Zhao and Li, 2006; Astikainen and Hietanen, 2009; Maekawa et al., 2009; Li et al., 2012); others have introduced a target detection visual task unrelated to vMNN stimuli (counting targets in Chang et al., 2010; button presses as a response to the target: Tales et al., 1999,^2008^; Tales and Butler, 2006). Sometimes the target is presented in the center of the visual field, while standards and deviants appear more peripherally (e.g., in Kremláček, 2006; Stefanics et al., 2012; Kuldkepp et al., 2013).

Due to the fact that the (v)MMN has clinical value (e.g., Tanaka et al., 2001; Horimoto et al., 2002; Tales et al., 2002,^2008^; Lorenzo-López et al., 2004; Tales and Butler, 2006; Hosák et al., 2008; Urban et al., 2008; Kenemans et al., 2010; all recently reviewed together with MMN studies by Näätänen et al., 2011,^2012^), it certainly calls for rigorous and standardized measurement procedures. Furthermore, a systematic look at this clinical work also reveals the same important discrepancy (i.e., difficulties in controlling one’s attention) between auditory and visual MMNs. For example, there are reports on two generators of the MMN in the auditory modality – one being a more perceptual feature-related, supratemporal generator and the other a cognitively higher, attention-switching frontal generator in nature (Giard et al., 1990). In vision, the vMMN has mainly been discovered in parieto-occipital and occipito-temporal and only seldom in frontal sites (Wei et al., 2002; Astikainen et al., 2008; Hosák et al., 2008). However, recently Kimura et al. (2010),^2011^ proposed the existence of two overlapping vMMNs: a more posterior sensory vMMN reflecting refractoriness and N1 and a more fronto-central cognitive or memory-dependent vMMN. The original oddball design for measuring the MMN with 10–20% of deviant signals has great value as a clean experimental procedure but, at the same time, it is very time-consuming. This is the main reason why Näätänen et al. (2004) developed a new paradigm (“Optimum 1”). Optimum 1 is a multi-feature paradigm that allows recording of multiple MMNs in a session with four to five deviants, 10–12.5% of each. The deviants differ from the standard in one feature, and can be presented alternately with the standard stimulus. Recently, Fisher et al. (2011) modified this paradigm by making it shorter and showing that three deviants (frequency, intensity, and duration of the sound) also elicited attenuated MMNs that were still of a reasonable size. As far as we know, the optimal multi-feature paradigm has not yet been applied in the context of visual automatic change detection (vMMN). Still, some researchers (Zhao and Li, 2006; Astikainen and Hietanen, 2009) have successfully presented two deviants (fearful or sad and happy faces) equiprobably in the same session (5 or 10%, for these studies, respectively). This is a very close approximation to the simple form of the optimum design. However, these authors did not compare their results to oddball data that, we believe, is worth of doing.

Obviously, we cannot easily stop participants from blinking but we can help their brain by presenting stimuli that are difficult to ignore or that are even considered to be processed automatically, like movements (Gibson, 1950) or faces (Palermo and Rhodes, 2007). In this study, we deal with schematic emotional faces for at least three reasons – *automaticity*, *simplicity* and *relevance*. (1) By automaticity, we mean that faces have often been reported as having been processed with high priority and without conscious effort and attention (Palermo and Rhodes, 2007). Contrary to many other objects, there is even a face-specific ERP component, N170, indicating fast detection of faces (Bentin et al., 1996). Studies also show that a basic categorization between a face and a non-face takes place in an even earlier time range (at about 100 ms, Pegna et al., 2004). Furthermore, probably due to the evolutionary processes, it is the expressional value of a face that most likely gets preferentially processed (Palermo and Rhodes, 2007). There have been several demonstrations that this automatic emotion processing of a face is asymmetric, favoring an angry or threatening face over a neutral or a happy one (Hansen and Hansen, 1988; Öhman et al., 2001; Weymar et al., 2011 with pop-out displays; Schupp et al., 2004; Stefanics et al., 2012) and involving the right hemisphere more than the left hemisphere (Palermo and Rhodes, 2007). Some studies with positive (happy) and negative (angry, sad, or fearful) faces as stimuli have reported longer latencies (Astikainen and Hietanen, 2009) but larger amplitudes for the negative face difference wave (Zhao and Li, 2006; but see also Xu et al., 2013 who found remarkable gender differences in the brain responses to schematic faces). Any emotion can be characterized by its valence and intensity (i.e., arousal value; Russell, 1980; Posner et al., 2005). These two categories tend to be temporally separated in their effects on ERPs (Olofsson et al., 2008). Olofsson et al. (2008) conclude in their review that the valence of stimuli is related to short latency (100–200 ms) ERPs, and arousal to longer-latency ERPs (200–300 ms). Thus, our brain differentiates between good and bad very quickly. However, sometimes happy face advantage is reported (e.g., Juth et al., 2005; Becker et al., 2011) attributing the effect to the communicative importance (Becker et al., 2011) or relative ease of perceptual processing of the happy face (Juth et al., 2005).

(2) The second reason for using schematic emotional faces as stimuli is their simplicity and controllability. It has been proven that simple schematic faces differing in only a few features (i.e., direction of the eyebrows and the mouth) but having no identity (i.e., does not likely resemble any real person) are rated as relatively natural and signaling different emotions (Horstmann, 2009). Horstmann’s article shows that, with respect to the ability to signal threat and being natural, the most optimal set of schematic faces is the one that Öhman et al. (2001) established. In a recent paper, Becker et al. (2011) also point out that a stimulus-set may often contain confounds (like white teeth in a happy face) explaining effects that have mistakenly been attributed to an emotion that can certainly be avoided with good schematic face-stimuli.
(3) Relevance refers to the fact that schematic faces have already been used as stimuli in vMMN research (Chang et al., 2010; Li et al., 2012). These studies, together with the ones with photographic faces (e.g., Astikainen and Hietanen, 2009; Stefanics et al., 2012) and those involving face-processing ERPs (i.e., the N170), provide the temporal frame of reference used later in the present study. Typical latencies for the vMMN are 200–320 ms (Li et al., 2012), 100–350 ms (Chang et al., 2010), 150–180 ms, and 280–320 ms (Astikainen and Hietanen, 2009), 170–360 ms (Stefanics et al., 2012), 110–360 ms for happy and 120–430 ms for sad faces (Zhao and Li, 2006). Another aspect of relevancy comes from a recent study where the preferential processing of an angry face was present with both, schematic and photographic stimuli (Lipp et al., 2009).

In the current study, we measured vMMN for schematic faces (happy, angry and neutral) in two different designs: the traditional oddball and a variant of the optimal (or “optimum” - these two terms will be used alternately here) multi-feature paradigm. We expect to (1) measure vMMN for schematic faces in posterior and probably also frontocentral sites; (2) find angry-face superiority (i.e., earlier or stronger response) in eliciting vMMN and (3) demonstrate that the multi-feature optimum paradigm can also successfully replace the traditional oddball paradigm in the visual domain. We also look at relations between subjective ratings in stimuli and vMMN, but it remains rather descriptive.

## MATERIALS AND METHODS

### PARTICIPANTS

Participants were eleven volunteering students (mean age 23.1 years, SD = 3.7 years, six women). They all had normal or corrected-to-normal vision and were right-handed. The study was approved by the Research Ethics Committee of the University of Tartu and the participants signed a written consent.

### STIMULI AND PROCEDURE

**FIGURE 1:**
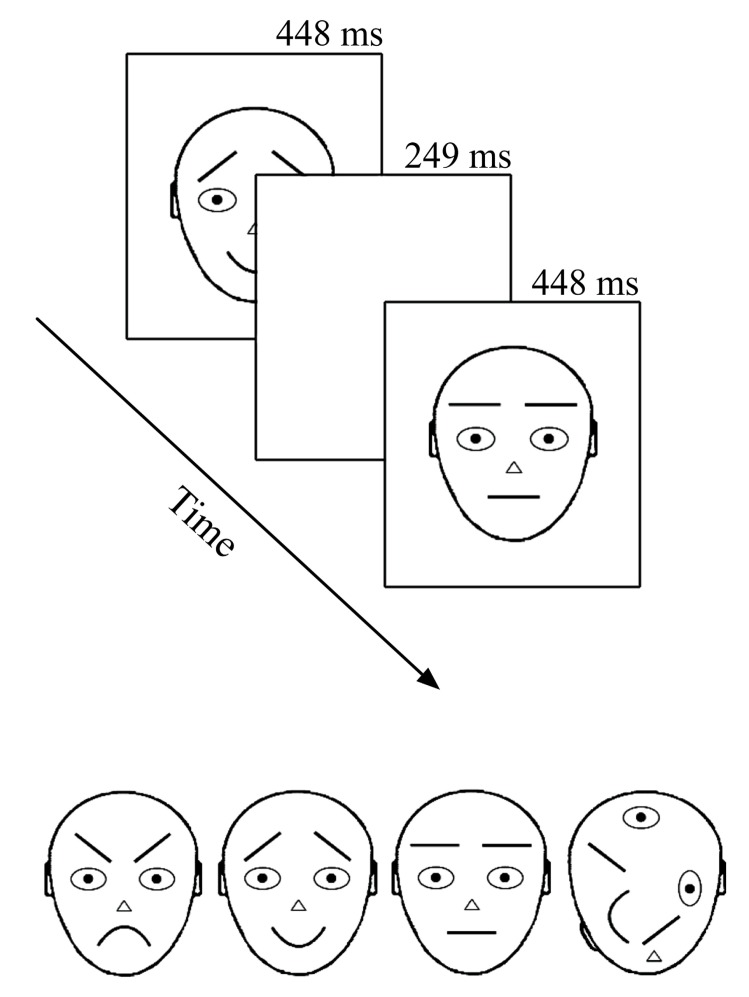
**Stimuli and presentation of stimuli.** In the oddball experiment only upward stimuli were used. In the optimum, the stimuli were also rotated by 180°. Stimuli are labeled from left to right: angry, happy, neutral, and target.

Recording took place in an electrically shielded semi-darkened chamber. The presentation screen (*Mitsubishi Diamond Pro 2070SB*, 22”; 60 Hz) was looked through a window at a distance of 114 cm. Stimuli were presented under the control of a Matlab program (MathWorks, Inc., Natick, MA, USA) for 249 ms with a 448 ms offset-to-onset interval (i.e., ISI = 448 ms) in the center of the computer screen (see **Figure 1** upper panel). The relatively long presentation time was chosen according to previous literature with comparable intervals (Astikainen and Hietanen, 2009; Stefanics et al., 2012; Kimura and Takeda, 2013) and pre-testing where shorter on-time rates tended to distress participants. During the ISI, the screen remained white (same as the stimulus background, 112 cd/m^2^). Stimuli were black-line schematic faces (674 × 789 pixels, i.e., 10.5° × 13.5°) on a white background (luminance 112 cd/m^2^) – neutral, angry, and happy plus a non-face object with scrambled face-like elements (adapted from Öhman et al., 2001 by Kukk, 2010; see **Figure 1** lower panel). The remarkable size of stimuli warranted that when observers looked at the center of the screen (as were the given instructions), important parts (i.e., mouth and eyebrows) of the to-be-ignored schematic faces appeared outside the fully attended foveal area. At the same time, a foveal part of the target stimulus (T) was optimal for its detection and no extra eye movements or search was needed.

All participants took part in two experiments – one with an oddball design and the other with a variant of optimum design. The sequence of experiments was pseudo-random and there was typically about 1.5 years between the measurements.

#### ODDBALL DESIGN

There were four different conditions in the experiment with an oddball design. For calculating the vMMN, the conditions consisted of following standard (S) and deviant (D) combinations: (1) angry D – neutral S; (2) happy D – neutral S; (3) neutral D – angry S; and (4) neutral D – happy S. In addition, we presented a non-face object as an attention-capturing target stimulus for each condition. All stimuli were presented in an upright position (as illustrated in **Figure 1** lower panel). In all four conditions, stimuli were arranged into 30 blocks, each consisting of 37 stimulus presentations. The first five presentations were always standard stimuli and thereafter standard, deviant, and target stimuli appeared pseudo-randomly. The pseudo-random sequence in the oddball experiment followed some simple rules: the overall proportion of both, deviants, and targets was 12.5% each and the minimum number of consecutive standards was two. This resulted in 120 deviant stimulus presentations per condition.

#### OPTIMUM DESIGN

There were three different conditions in the experiment. In all of them, stimuli were arranged into 40 blocks that consisted of 37 stimulus presentations (as in an oddball experiment). The first five presentations were always standard stimuli and thereafter standards appeared alternately to deviant or target stimuli (meaning that every second stimulus was a standard). In our variant of the optimum design, one of the schematic faces (either neutral, angry, or happy, see **Figure 1** lower panel) was the standard stimulus in each of the three conditions. Standard stimuli were always presented in an upright position. The deviant stimuli were, depending on the condition, two remaining schematic faces and their inverted versions (180° rotated, not illustrated in **Figure 1**, and not analyzed in the current article), inverted version of the standard stimulus and standard stimulus presented is a position of a deviant (both not analyzed here). Altogether, the proportion of six different deviants (including standard presented as a deviant) was 37.5%, which resulted in 80 presentations per one deviant per condition. As in an oddball experiment, the scrambled non-face object was always presented as a target (here again, either upward or inverted) with the proportion of 12.5%. Participants were, again, instructed to ignore all the other stimuli and press the mouse key with their right hand as quickly as possible whenever the target appeared on the screen. As already told, targets were easily detectable and there was no obvious reason to attend to standard and deviant stimuli. The onset of the blocks consisting of 37 presentations was self-initiated by the participant in both experiments. The idea behind the block-wise setup was to help participants follow the instruction to avoid blinking and body movements during the recording and to compensate for the effort during these self-terminated breaks.

### EEG MEASUREMENT AND DATA ANALYSES

The electroencephalogram (EEG) was recorded using a system of 32 active electrodes (Active Two, BioSemi, Amsterdam, Netherlands). In accordance with the 10/20 system, the recording sites were FP1, AF3, F7, F3, FC1, FC5, T7, C3, CP1, CP5, P7, P3, Pz, PO3, O1, Oz, O2, PO4, P4, P8, CP6, CP2, C4, T8, FC6, FC2, F4, F8, AF4, FP2, FZ, and Cz. Two additional electrodes were placed behind the earlobes of the participant and their signal was offline used as a reference. Additional electrodes were placed below and under the left eye (to record vertical eye movements and blinks) and to the outer canthi of the eyes to monitor horizontal eye-movements. The EEG was online registered with a sampling rate of 1024 Hz and a band pass filter of 0.16–100 Hz.

EEG data were offline analyzed with Brain Vision Analyzer 1.05 (Brain Products GmbH, Munich, Germany). A moderate 1–30 Hz filter (24 oct/dB) was applied to the data. Data were segmented into 950 ms pieces around the stimulus presentation (from -150 ms pre-stimulus to 800 ms post-stimulus) and a 100 ms pre-stimulus period was used as a baseline correction interval. A built-in ocular correction was also applied (Gratton et al., 1983). Artifact-rejection criteria for any segment was applied as follows: (1) maximal amplitude difference between consecutive data points over 50 μV; (2) maximal allowed amplitude difference of 100 μV; (3) amplitudes over 100 μV and below -100 μV; and (4) no more than 100 ms of low activity (0.5 μV). For the subsequent data analysis, electrodes were pooled together: O1, O2, and Oz for the occipital area (O), P4, P8, PO4, and Pz for the right parietal (RP), P3, P7, PO3, and Pz for the left parietal (LP); P3, P4, P7, P8, PO3, PO4, and Pz for the parietal activity (P), Cz, FC1, and FC2 for the midfrontal (MF), AF3, F3, and Fz for the left frontal (LF), AF4, F4, and Fz for the right frontal (RF) and AF3, AF4, F3, F4, and Fz for the frontal (F) activity.

To equalize the number of trials under comparison between standard and deviant stimuli, the respective proportion of trials was randomly selected out of each set of standards. It was visually checked that the selection did not influence the general shape of the averaged standard stimulus waveform. To analyze the individual data, standard, deviant and difference (vMMN) waveforms for each observer (*N* = 11), design (oddball and optimum) and condition (four different conditions in an oddball and three in an optimum experiment, see Stimuli and Procedure) were exported in ASCII format. Statistical comparisons were conducted in Statistica 8.0 (StatSoft Inc., Tulsa, OK, USA). Repeated measures analysis of variance (ANOVA) with a Greenhouse-Geisser correction of degrees of freedom (applied when needed for electrophysiological comparisons) and Tukey HSD or Bonferroni test for post-hoc comparisons was conducted.

We characterized MMN by a maximal negative peak (determined as the mean value of three points: the peak and its neighbors) and a mean amplitude within five predefined intervals (based on the literature and visual inspection of results).These intervals (100–140, 140–180, 180–260, 260–340, and 340–500 ms) are rough estimations of the latency of the vMMN. However, the mean linear product-moment correlation between the highest negative peak and the average activity for the angry or the happy face difference waves (over all observers, conditions, pooled electrode sites and five intervals) was 0.961 (ranging from 0.943 to 0.971). Thus, these two estimates of the vMMN are very similar to each other. Derived from that, we used only the average amplitude within the interval in further analyses.

### BEHAVIORAL RECORDINGS AND DATA ANALYSES

The participants’ manual reactions (as indicated by mouse key presses) were online recorded in milliseconds. Data were offline analyzed in Statistica 8.0 (StatSoft Inc., Tulsa, OK, USA) for calculating target detection probabilities and mean reaction times (RTs) for detections, as well as conducting comparisons between optimum and oddball designs using paired t-test for dependent samples.

### POST-EXPERIMENTAL QUESTIONNAIRE AND DATA ANALYSES

After the experiment, a short inventory was conducted asking participants to rate on a nine-point scale how (1) positive (1) or negative (9); (2) calming (1) or arousing (9); and (3) attention-attracting (1– ignored or unnoticed, 9 – irresistible) each of the stimuli had subjectively been felt. Participants were also asked to label stimuli and to describe their strategy (i.e., verbal or figurative, not analyzed here) used in the experiment. Subjects’ ratings to each question were coded into values from one to nine and mean values of each category (valence, arousal and attention-attracting) were calculated. Repeated measures analysis of variance (ANOVA) was used to calculate mean differences in participants subjective answers to each stimuli and category. Also, answers after participating in an experiment with optimum design were compared to answers after participating in an experiment with oddball design (this was done using ten participants’ data, because one of the participants conducted both experiments on the same day and filled the questionnaire once). During the experimental session labeling of stimuli was consciously avoided, by showing drawings of stimuli and calling them “it” or “this” when instructions were given.

## RESULTS

### BEHAVIORAL AND POST-EXPERIMENTAL QUESTIONNAIRE’S RESULTS

Altogether, the detection of targets was performed very well. The detection probability was remarkable being 96.5% for the oddball and 92.3% for the optimum sessions. Also RTs for detections –395.0 ms (SD = 45.59 ms) and 405.0 ms (SD = 41.7 ms) for the oddball and the optimum experiment, respectively – were similar in both experiments (*t*(10) = 1.41, *p* = 0.188).

One may ask whether such simple stimuli carry any emotion-related meaning at all. We first tested if there were differences in rating the stimuli depending on whether it was done after an oddball or an optimum experiment. A 2 (condition: oddball and optimum) × 4 (stimulus: angry, happy, neutral, and face-like object, i.e., target) repeated measures ANOVA showed that the subjective ratings of valence, arousal and attention did not differ from each other when compared between the two conditions [*F*(1,9) = 4.494, *p* = 0.063 for valence, *F*(1,9) = 0.429, *p* = 0.529 for arousal and *F*(1,9) = 0.243, *p* = 0.634 for attention]. This allows us to use all questionnaire results together in further analyses. **Table 1** shows the mean ratings of the stimuli according to two intrinsic dimensions of emotions (valence and arousal) as well as how much the stimuli had caught the attention of participants.

**Table 1:** Mean subjective ratings of stimuli (repeated measures ANOVA).

Stimulus						
	Angry	Happy	Neutral	Target	*F*(3, 30)	*p*
Valence	8.68(0.63)^HNT^	2.91(2.30)^AN^	5.14(1.40)^AH^	4.09(1.7)^A^	34.71	<0.0001
Arousal	6.36(1.52)	5.00(1.36)	4.55(1.56)	4.68(1.77)	2.92	0.05
Attention	6.18(2.19)	5.36(1.98)^T^	5.18(1.52)^T^	7.68(1.38)^HN^	5.06	0.006

*Notes. *N* = 11. SD is in parentheses. ^A, H, N^ and ^T^ refer to differences (Bonferroni *post hoc* test, *p* < 0.05) in the angry, happy, neutral, and target stimulus, respectively. The scales for valence were valence 1 – positive and 9 – negative, for arousal 1 – calming and 9 – exciting, and for attention 1 – unattended, ignored and 9 – fully attended, irresistible.*

As can be seen, target and neutral faces were indeed perceived as neutral, whereas angry and happy faces were perceived to be negative and positive, respectively. Arousing value of the stimuli did not differ significantly from each other. With respect to attention, targets were more attention capturing than the happy and the neutral stimuli but the angry stimulus was perceived to be equally attention catching as compared to the target. This speaks for the subjective superiority of the angry (as a social threat-carrying) stimulus as a perceptual object. It is of interest that the automatic attention allocated to the target tended to relate negatively to the attention paid to the angry (correlation being -0.618, *p* < 0.01).

### TOPOGRAPHY OF THE ERP DIFFERENCE (VMMN)

To see whether there are lateralisation effects, we first compared responses in frontal, parietal and occipital left and right locations (LF, RF, LP, RP, O1, O2). This was done by ANOVA (mean amplitude of the difference waves in five intervals as repeated variable) and with lateralisation (left or right), position (frontal, parietal, and occipital), condition (oddball, optimum) and stimulus (angry, happy) as factors. We found no lateralisation main effect [*F*(1, 240) = 1.185, *p* = 0.2774] nor any interaction with these factors indicating basic symmetry in the brain responses. Mean amplitude of the differential response did differ between 100 and 500 ms as there was a main effect of interval [*F*(2.42, 581.63) = 39.111, *p* < 0.00001, η^2 ^= 0.140, ε = 0.606]. Instead of negativity, the latest interval (340–500 ms) showed pervasive positivity (0.229 μV) and the activity differed from the mean activity of all the other intervals (being, starting from the earliest -0.765, -1.058, -1.013 and -0.781 μV, respectively; all the differences confirmed by the Tukey post-hoc test). Similar analysis did not indicate any difference between frontal and midfrontal pooled sites. Thus, in further analyses we compare only central pooled positions (F, P, and O) to each other (if not specified differently).

**FIGURE 2:**
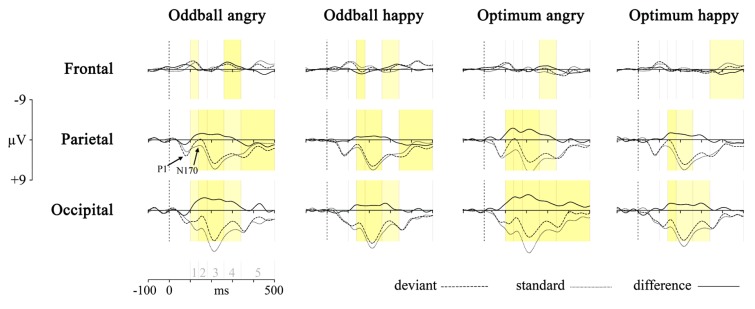
**Processing of deviant angry or happy stimulus in the oddball (two left panels) and optimum (two right panels) experiment as compared to processing of the same stimulus as standard in the other experimental session.** The dotted line is the averaged activity when processing of the standard, the broken line is for the deviant and the solid line is the difference wave (vMMN, deviant – standard). Graphs represent results from three pooled sites (frontal, parietal, and occipital). Shaded areas represent the difference between processing of deviant and standard: darker yellow is for full-interval difference and lighter yellow is for the significant difference between deviant and standard within more than half of the interval. Intervals under comparison are marked in the scale in the lower left corner: 1 – 100–140 ms, 2 – 140–180 ms, 3 – 180–260 ms, 4 – 260–340 ms, and 5 – 340–500 ms.

First, the responses to deviant stimulus, either angry or happy one, were compared to the responses to the same stimulus presented as a standard in another session (i.e., Angry-Deviant-minus-Angry-Standard and Happy-Deviant-minus-Happy-Standard, see also Stefanics et al., 2012 for comparison of physically identical stimuli). This was done for the oddball and optimal experiments. The resulting difference waveforms as well as standard and deviant waveforms are presented in **Figure 2**.

Processing of the same stimulus as deviant and standard was subjected to an unpaired point-by-point *t*-test (Vision Analyzer 1.05, Brain Vision) with a rather conservative criterion, *t* < -5 or *t* > 5. Significant *t*-values are marked on the waveforms in **Figure 2** with two colors indicating how much time within the interval the processing of these two stimuli differed from each other. It is seen that there are reliable differences between deviant and standard waveforms in 140–340 ms posteriorly, most likely representing the vMMN response. This is another reason, together with ANOVA results reported above, to refine most of our analyses to the midlatency intervals (140–180, 180–260, and 260–340 ms) where the vMMN is most likely elicited.

Although the pattern is not fully clear, is can be seen that the negativity presumably representing vMMN is the most consistently present at occipital sites. This processing negativity is pretty extended in time possibly comprising also attention-related processing. It can be seen that processing of happy stimulus shows a remarkable similarity between experiments. Another common feature of the processing is the widespread emerging positivity after 340 ms. Activity in this late time interval may refer to attended or conscious processing of stimuli. At the same time, there is also a frontal vMMN as frontal negative deflection is shown in the 260–340 ms time-interval in the oddball paradigm for both stimuli, and also for the angry in the optimum paradigm. This explains why we included frontal pooled site into further analyses.

### ODDBALL VS. OPTIMUM

One of the central aims of the study was to compare vMMNs to the schematic faces elicited in the two different MMN-paradigms, the classical oddball and a variant of the optimum, with each other. Next, differential processing of the deviant stimulus, either angry or happy one (i.e., Angry-Deviant-minus-Angry-Standard and Happy-Deviant-minus-Happy-Standard) was inspected more closely. The occipital, parietal and frontal sites were selected for the graphical presentation because they showed, also at the individual level, the most prominent negative amplitudes (see **Figure 2**). Such a selection was supported statistically, too.

In a repeated measures 3 (electrode sites: F, P, and O) × 3 (temporal intervals: 140–180, 180–260, and 260–340 ms ) × 2 (angry vs. happy stimuli) × 2 (condition: optimal and oddball) ANOVA ) it was revealed that pooled electrode site had an main effect on results [*F*(1.15, 11.53) = 15.17, *p* < 0.01, η^2^ = 0.603, ε = 0.577 ]. At the same time, neither experimental paradigms (oddball vs. optimal), stimuli (angry vs. happy schematic face) or time differed from each other: [*F*(1, 10) = 0.147, *p* = .710, η^2^ = 0.015; *F*(1, 10) = 3.023, *p* = .113, η^2^ = 0.232 and *F*(2, 20) = 0.492, *p* = 0.618, η^2^ = 0.047, respectively]. Tukey post-hoc test confirmed that posterior sites (P, O) had more negative amplitudes than the frontal one (F). No interaction between these factors was significant. vMMNs obtained at the occipital, parietal and frontal sites are plotted in **Figure 4**. Although visually somewhat different, mean amplitudes for these four vMMN curves do not differ from each other.

It again confirms that at mid-latency the oddball and the variant of optimal paradigm give relatively similar estimations of the MMN.

### CONTROL FOR FEATURE DIFFERENCES AND REFRACTORY EFFECTS

In addition to the between-series difference waveforms analyzed so far (Angry-Deviant-minus-Angry-Standard and Happy-Deviant-minus-Happy-Standard) we also found classical, within-series difference waves (Angry-Deviant-minus-Neutral-Standard and Happy-Deviant-minus-Neutral-Standard). Again, a 2 (type of standard: type of deviant) × 3 (recording site: F, P, O,) × 2 (stimulus: angry or happy face) × 3 (temporal intervals) × 2 (oddball and optimum) ANOVA indicated that type of standard (physically the same or different from the deviant) did not matter for the generation of difference waves [*F*(1, 10) = 2.419, *p* = 0.1509, η^2^ = 0.195]. Thus, it did not make any difference whether deviants were compared to physically identical or different standards. As the vMMN is also thought to represent activity from fresh units encoding new input (i.e., the refractoriness issue; see Kimura, 2012), this result refers to the fact that the contamination of the vMMN with the refractory reactions is non-fatal. However, an emerging interaction between the stimuli (angry or happy) and the comparison (either with the same or different standard) [*F*(1, 10) = 6.4371, *p* = 0.0295 η^2^ = 0.392] indicated that stimuli may differ in this respect. We found that processing of happy stimulus was vulnerable to the standard stimulus similarity showing only about half of the amplitude in the same standard condition as compared to the neutral standard condition (-0.76 vs. -1.35 μV).

In MMN research, there is always the question of, what is behind the difference waveforms. The very first candidate is a physical difference between standard and deviant stimuli that would result in a larger amplitude of the N1 component and an earlier MMN (see Kimura, 2012). Next, we analyzed data from two inverse conditions, i.e., Angry-Deviant-minus-Neutral-Standard will be compared to Neutral-Deviant-minus-Angry-Standard and Happy-Deviant-minus-Neutral-Standard will be compared to Neutral-Deviant-minus-Happy-Standard. This is typically – in the case of equal sized MMNs – considered to help control for exogenous effects in the MMN. For this we conducted a 2 (type of standard: emotional or neutral schematic face) × 3 (localization site: F, P, O) × 2 (stimulus: angry or happy face) × 3 (temporal intervals) repeated measures ANOVA (with experimental design as the grouping factor). The mere direction of comparison did not have a significant effect on average activity in the intervals [*F*(1, 10) = 3.898, *p* = 0.077, η^2^ = 0.281]. However, the results show an interaction between pooled electrodes and comparison direction (i.e., whether emotional stimulus is compared to the neutral or vice versa) [*F*(1.54, 15.44) = 13.165, *p* = 0.001 η^2^ = 0.568, ε = 0.772]. A Tukey post-hoc test confirmed that emotional deviants had more negativity at occipital and parietal recording sites compared to the respective neutral deviants. Thus, it appears that conducting a standard-deviant inverse procedure has the built-in risk that such comparison does not work, and even does not have to work. We believe that our current case belongs to the latter category – emotional deviant stimulus just gets an extra processing because of its evolutionary significance. Similar pattern for an angry face was found with a search task using direct and averted gaze direction: a face with direct gaze, indicating more threat, was more efficiently found among angry faces with averted gaze than vice versa (von Grünau and Anston, 1995).

The rare emotional stimulus (either angry or happy deviant) among neutral standards was obviously more salient and attracted more automatic processing resources than the neutral deviant among emotional standards. Actually, this was what we implicitly expected when choosing emotional to-be-ignored stimuli! Thus, we failed to test the feature equality between stimuli but, at the same time, found some proof that emotional deviants attract automatic attention. In the following analyses we abandon the reversed neutral deviant conditions.

### IS THERE ANY ANGRY ADVANTAGE?

**FIGURE 3:**
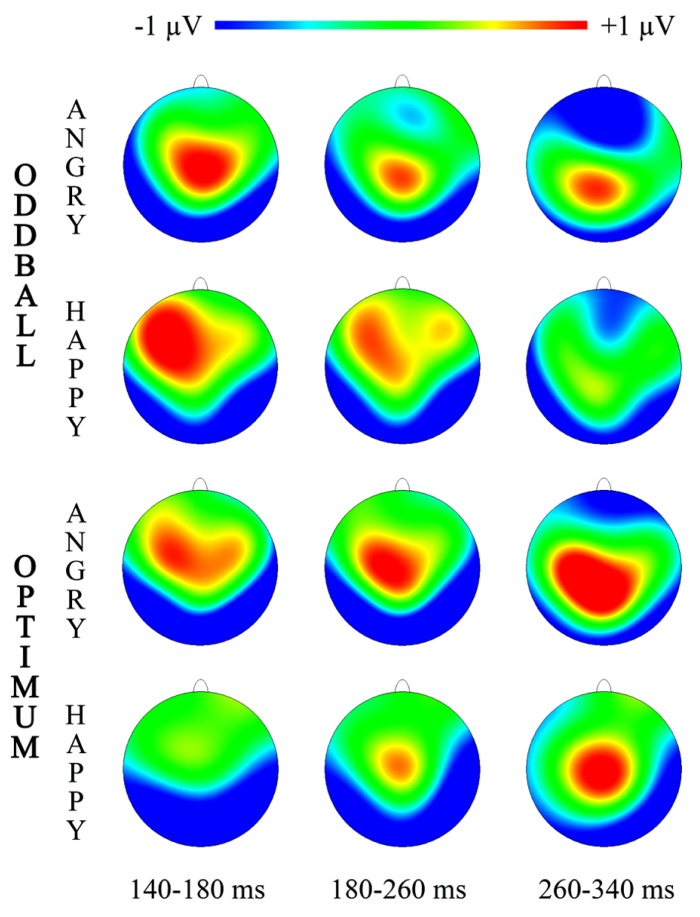
Topographic maps for three intervals of vMMN representing “Angry-Deviant-minus-Angry-Standard” and “Happy-Deviant-minus-Happy-Standard” activity for the oddball and optimum paradigms.

**FIGURE 4:**
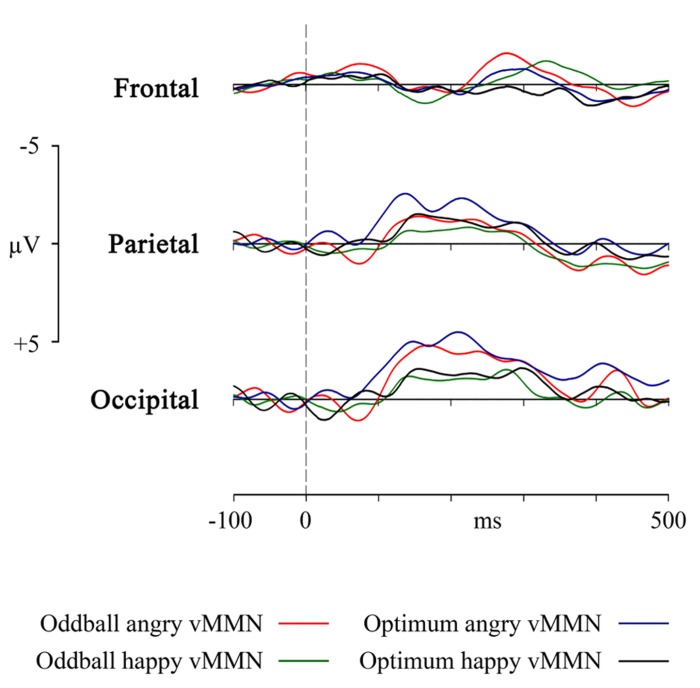
VMMN as a response in frontal, parietal and occipital pooled sites to the angry and happy schematic face (oddball and optimum paradigms).

According to our analyses, the quick answer to this question appears to be “no”. Still, **Figures 2, 3**, and **4** describe at least some differences between the two deviant emotional stimuli with the opposite valences. Also, previous analyses indicate some advantages in processing of the angry stimulus as compared to the happy one. For example, the findings showing that (1) the angry stimulus got subjectively more attention than other non-targets (**Table 1**); (2) in case of the angry stimulus processing negativity started earlier than for the happy deviant (**Figure 2**); or (3) processing of the angry face did not depend on the standard stimulus, all indicate some superiority of the angry face for perception.

Topographical illustrations of visual MMNs are plotted in **Figure 3** for three time-intervals where more prominent significant differences between deviant and standard processing were shown (**Figure 2**).

Furthermore, it may be logical to ask whether the theoretically plausible superiority of the angry face that seems to be present in the **Figure 4** survives statistical testing. Main effects ANOVA (2 stimuli × 2 designs × 3 electrodes) for the mean activity in the interval of the most prominent vMMN (140–180 ms) shows that there is again the already reported main effect of electrode [*F*(2, 127) = 23.11, *p* < 0.00001, η^2^ = 0.267] and also a main effect of stimulus [*F*(1, 127) = 4.494, *p* = 0.028, η^2^ = 0.038]. Generally, within this interval (and also in the next interval), angry stimulus produces vMMNs with higher average amplitude but this does not depend on the experimental design.

Altogether, although we were unable to discover a broad and striking angry superiority effect at the level of deviance detection in the brain there are some allusions to it.

### VMMN’S RELATION TO SUBJECTIVE RATINGS OF STIMULI

Finally we examined whether individual ratings of each stimulus valence, arousal and power to attract one’s attention were related to the mean amplitude of the difference wave (vMMN) within the five intervals (100–140, 140–180, 180–260, 260–340, and 340–500 ms) and extended list of pooled electrode positions (F, LF, RF, MF, P, LP, RP, O). We decided to use wider range of positions and temporal intervals here because it may be meaningful for emotional attention issues. Instead of single correlations we ran multiple regression analyses (forward stepwise method) to predict subjective ratings from mean amplitudes of the difference waves (vMMNs) in all five intervals. For valence and arousal ratings and the attention that was subjectively allocated to the happy stimulus or to the target the models either did not converge or reduce the set of predictors effectively enough. For the angry stimulus there appeared to be a set of independent predictors accounting for 71% of attention subjectively paid to it. A significant model was achieved [*F*(11, 9) = 5.46, *p* < 0.00836] with an adjusted *R*^2^ = 0.710 with following significant predictors and standardized regression coefficients in parentheses: for 100–140 ms at LF (0.463), for 140–180 ms at RP (-0.670), MF (1.061), RF (6.930), F (-6.344), for 260–340 ms at LP (0.793), RP (-0.200), MF (0.981), RF (-3.340), F (2.152) and for 340–500 ms at MF(0.336). A closer look at all these 11 predictors reveals some patterns: (1) most of them are located frontally (MF, F, RF); (2) there are only two typical predictor intervals: 140–180 ms and 260–340 ms; (3) two out of three posterior predictors (LP, RP) are in 260–340 ms, and (4) more predictors lie in the right than in the left.

## DISCUSSION

Our results show that (1) in the occipital and parietal area, the oddball and the optimum designs elicit vMMN equally in automatic deviance detection; (2) emotional faces are more efficient in eliciting vMMN in the brain than the neutral schematic face; (3) automatic visual change detection is the most powerful during 140–260 ms after stimulus onset and at the posterior (P, O) sites; (4) although participants were asked to ignore it, the angry stimulus catches as much subjective attention as the target (**Table 1**, **Figure 2**); and (5) despite the differences in subjective ratings of valence and attention-catching, the angry and the happy deviant stimuli do not differ much from each other, but both differ from the neutral stimulus in processing at the brain level; (6) allocation of attention to the angry stimulus was hard to avoid.

### DID WE REGISTER THE VMMN?

At first we should make clear whether we dealt with the vMMN at all. The general shape of the vMMN tends to vary a bit along with stimulus and experiment type. Our stimuli – the sequence of schematic or realistic faces – resembled the ones used in several previous studies (Zhao and Li, 2006; Astikainen and Hietanen, 2009; Chang et al., 2010; Stefanics et al., 2012).The shapes of the deviant-minus-standard difference waves obtained, and their prominently posterior location were comparable as well. Our relatively early posterior vMMN also includes at least some N1 and refractory activity (e.g., Kimura et al., 2010,^2011^; Kimura, 2012; Kimura and Takeda, 2013). Inspired by the adaptation vs. memory trace debate on the MMN (e.g., Näätänen et al., 2005), we argue that the adaptation-part (i.e., difference in N1) is not the most decisive nor the only factor here because: (a) the observed negative differential posterior activity lasts for about 200 ms (140–340 ms) that is too long and too late for the pure early sensory activity (indicated by P1 and N170, see **Figure 2**); (b) the afferent activity should depend on the physical difference between deviant and standard (that could have been either neutral stimulus of the same session or angry/happy face of another session in our study) but the posterior vMMNs we found with these two types of standards, differed only for the happy not for the angry deviant. This discrepancy may be related to the amount of automatic attention the angry stimulus inevitably catches.

Due to the nature of stimuli it was very difficult to avoid automatic attention allocated to them. Our stimuli were presented in the center of the visual field for relatively long time (250 ms) to allow attentional processes to operate. At the same time, our stimuli extended over relatively wide area (13.5° × 10.5°), so that informative parts of them (mouth, eyebrows) were certainly not presented foveally but rather processed automatically. Attention was allocated more to the target and angry face than to other stimuli (**Table 1**). At the same time, extra involuntary attention to the angry stimulus did not yield any considerable differences in ERPs at the vMMN interval up to 260 ms. Of course, **Figure 2** shows that angry face tends to elicit difference in processing deviant and standard also around the N1 range (100–140 ms) that refers to the role of attention in detecting them. After posterior vMMN there was some frontal vMMN in processing of the angry face (see **Figures 2** and **3**, 260–340 ms) probably reflecting the automatic attention switch (Giard et al., 1990). A probable attention reorienting was supported by the multiple regression analysis showing that the vMMN in the EPN range (Schupp et al., 2006; Olofsson et al., 2008) was related to the amount of attention allocated to the target. However, only a few positions (LP and RP, both 260–340 ms) were actually posterior. At the same time, this activity was lateralised being more in the right than in the left hemisphere (also observed by Stefanics et al., 2012). At the same time, these earlier posterior and later frontal difference curves did not differ between conditions and stimuli, probably due to a modest sample size. According to Kimura et al. (2010) and Kimura and Takeda (2013) the later and more anterior vMMN is even a more genuine marker of the automatic difference processing than the vMMN recorded from the more sensory areas.

### ODDBALL VS. OPTIMUM PARADIGM

The most important practical result of the study is the essential equivalence of the oddball and the optimal multi-feature design in eliciting the posterior vMMN. Experimental design did not have any main effect on any comparisons we performed. But let us take an intuitive look at **Figure 4**, representing the four vMMNs in the occipital, parietal and frontal area. Intuition tells us that in the optimum design, the processing of the angry deviant would elicit a higher amplitude vMMN than the happy deviant. This is close to the truth as in the restricted intervals the angry stimulus was processed with higher mean activity but this was a rather pervasive tendency at posterior sites, not specific to the oddball or optimum paradigm. It may be asked whether the ability not to discriminate between deviant stimuli is an advantage or a disadvantage of the oddball and optimum design. Probably the stimuli we used were not strong enough to produce such differences. It really deserves further investigation, but the encouraging fact is that the designs were rather equal and can be used intermittently, depending on specific needs.

However, it should be mentioned that the presentation probability for a single deviant in the oddball experiment was about twice as high as in the optimum experiment. Thus, some amount of the vMMN in the optimum paradigm could have originated from its lesser refractory state (see Kimura and Takeda, 2013). In the future research the refractoriness in the optimum paradigm should be systematically studied.

Further studies should contrast these two designs with equally salient, more neutral stimuli enabling to also test endogeneity. A good candidate for such a feature is visual motion (see Kuldkepp et al., 2013) differing in direction, velocity and duration, for example. We consider the future development of the visual optimal paradigm for the vMMN measurement truly promising as this would considerably facilitate its clinical implementation. Clinically applicable and standardized multi-feature vMMN experiments would be very welcome for diagnostic and treatment-monitoring purposes, for example in the case of Alzheimer’s disease and mild cognitive impairment (e.g., Tanaka et al., 2001; Tales et al., 2002,^2008^; Tales and Butler, 2006), schizophrenia (Urban et al., 2008) and alcohol intoxication (Kenemans et al., 2010).

### ANGRY VS. HAPPY FACES

The specific nature of the deviants – either angry or happy – did not explain the obtained vMMN waveforms. The most meaningful result was the general higher mean activity for the angry than the happy deviant but this was only seldom statistically significant. As other vMMNs for these two stimuli did not differ significantly, the registration of the vMMN was, indeed, relatively attention-free. Generally, the subjective state of the participant was expected to relate to vMMN amplitude (evidence reviewed in Näätänen et al., 2011). The fact that the valence of stimuli did not relate to the vMMN may be connected to the relatively late temporal window under close investigation. For the face, positive–negative categorization may take place even earlier than 100 ms (Palermo and Rhodes, 2007). The averaged vMMNs (**Figure 2** left most panel) are not too encouraging in this respect. The face-specific component N170 was found at around 160 ms (see **Figure 2**) and it did not differ between angry and happy stimuli. Neither did arousal (indicated by the subjective ratings). Our expected angry stimulus advantage (Öhman et al., 2001) or negativity bias (Stefanics et al., 2012) has been shown to have considerable gender differences (Bradley and Lang, 2007) that should be taken into account in future research (Xu et al., 2013).

### LIMITATIONS AND STRENGTHS

Our study is quite exploratory but we have raised several important issues that need a different study with finer spatial and temporal resolution and probably also with a larger sample, to be addressed. A larger sample would enable us to have a closer look on gender differences in lateralization that have been recently reported (Xu et al., 2013).

On the other side, our study has the strength of using a within-subjects design giving us the certainty that differences between conditions and stimuli are not produced by different groups. Another aspect is the use of a repeated measures design with at least satisfactory quality of each individual data set. To conclude, we have taken an important and rather successful step toward the establishing of the optimum multi-feature registration procedure of the vMMN.

## Conflict of Interest Statement

The authors declare that the research was conducted in the absence of any commercial or financial relationships that could be construed as a potential conflict of interest.
